# Paroxysmal hemicrania: a retrospective study of a consecutive series of 22 patients and a critical analysis of the diagnostic criteria

**DOI:** 10.1186/1129-2377-14-26

**Published:** 2013-03-20

**Authors:** Sanjay Prakash, Pooja Belani, Ashish Susvirkar, Aditi Trivedi, Sunil Ahuja, Animesh Patel

**Affiliations:** 1 Department of Neurology, Medical College, O-19, doctor’s quarter, jail road, Baroda, Gujarat, 390001, India; 2 Department of Medicine, Medical College, Baroda, Gujarat, India; 3 Department of Psychiatry, Medical College, Baroda, Gujarat, 390001, India

**Keywords:** Chronic daily headache, Paroxysmal hemicrania, Hemicrania continua, Cluster headache

## Abstract

**Background:**

Paroxysmal hemicrania (PH) is a probably underreported primary headache disorder. It is characterized by repeated attacks of severe, strictly unilateral pain lasting 2 to 30 minutes localized to orbital, supraorbital, and temporal areas accompanied by ipsilateral autonomic features. The hallmark of PH is the absolute cessation of the headache with indomethacin. However, these all features may not be present in all cases and a few cases may remain unclassified according to the 2nd Edition of The International classification of Headache Disorders (ICHD-II) criteria for PH.

**Methods:**

Twenty-two patients were included in this retrospective observation.

**Results:**

We describe 17 patients, observed over six years, who fulfilled the ICHD-II criteria for PH. In parallel, we identified five more patients in whom one of the features of the diagnostic criteria for PH was missing. Two patients did not show any evidence of cranial autonomic feature during the attacks of headache. Another two patients did not fulfill the criteria for PH as the maximum attack frequency was less than five. One patient had an incomplete response to indomethacin.

**Conclusion:**

A subset of patients may not have all the defined features of PH and there is a need for refinement of the existing diagnostic criteria.

## Background

Paroxysmal hemicrania (PH) is a rare primary headache disorder. It is classified in group 3 of the 2nd Edition of The International classification of Headache Disorders (ICHD-II) along with other trigeminal autonomic cephalalgias (TACs). The characteristic features of PH are: (a) repeated attacks of severe unilateral, orbital, supraorbital or temporal pain lasting for 2–30 min; (b) at least one ipsilateral cranial autonomic feature; (c) attacks frequency > 5 per day for more than half of the time, (d) complete response to therapeutic doses of indomethacin. All four characteristic features are essential to satisfy the ICHD-II criteria for PH [1]. However, a few patients may have atypical presentation [2,3].

Herein, in a retrospective study, we report 17 patients who fulfilled the ICHD-II criteria for PH. In parallel, we identified a group of patients (5 patients) who met all criteria minus one (either absence of cranial autonomic features or headache frequency < 5 per day or complete response to indomethacin).

## Methods

We performed a retrospective chart review of all patients with a putative diagnosis of PH, seen in the neurology department at our institute from February 2006 to July 2012. The patients having a diagnosis of PH or PH like headache and with a minimum follow-up of 3 months duration were included in the study. We reviewed the charts of each individual. A few patients were interviewed by telephone to complete the follow-up. Exclusion criteria were: (a) a possible secondary PH; (b) patients who were never subjected for neuroimaging, as we did not rule out the possibility of secondary PH in these patients; and (c) a follow-up of < 3 months duration. The study did not require approval by the local ethics committee as per the local regulations for retrospective observation. The majority of patients were seen and examined by a neurologist (SP). The patients fulfilling the ICHD-II criteria for PH were tabulated. Data are presented as percentages or as arithmetic mean with SD. Student’s t-test was used to compare the continuous data between the subgroups. The chi-square test with Yates’s correction was used for categorical data. A p-value < 0.05 was defined as statistically significant. Patients with atypical features were tabulated separately.

## Results

A total of 28 patients were identified with a putative diagnosis of PH or PH-like headache. One patient was excluded because of the possibility of secondary PH (pituitary adenoma). One patient was never subjected to neuroimaging and was excluded from the final analyses. Two other patients were excluded because of the incomplete follow-up (< 3 months). Two patients were excluded because of the incomplete data entry. Finally, twenty-two patients were identified. Seventeen patients (77%) fulfilled the ICHD-II criteria for PH. Two patients (9%) did not show any evidence of cranial autonomic features. Two patients (9%) did not fulfill the criteria for PH as the maximum attack frequency was less than five. One patient (5%) did not show a complete response to indomethacin.

Epidemiological profiles, and clinical features of patients fulfilling the criteria for PH (17 patients) are noted in Table 1. Patients with atypical features (with no cranial autonomic features or headache frequency < 5 per day or incomplete response to indomethacin) were summarized separately in Table 2.

**Table 1:** Epidemiological and clinical features of patients with parosysmal hemicrania

**Parameters**	**(n-17)**
Age ( years)(mean, range)	38 ± 12.3, 21–60
Sex (M:F)	1:1.1 ( 8:9)
Duration of illness	
Mean (SD)	38.2 ± 38.1 months
Range	3 months-10 yrs
Median	18 months
Laterality, n (%)	
Rt	8 (47)
Lt	9 (53)
Course, n (%)	
Chronic	15 (88)
Episodic	2 (12)
Site of headache, n (%)	
Orbital\retroorbital	15(88)
Temporal	10 (59)
Frontal	10 (59)
Parieto-occipital	7 (41)
Infra- orbital /maxillary	4 (24)
Peri auricular	2 (12)
Neck	2 (12)
Character of pain	
Throbbing	9 (53)
Non throbbing	12 (71)
Both throbbing and non throbbing	4 (24)
Severe or very severe	15 (88)
Interictal pain	
Present	8 (47)
Intermittent	7 (41)
Continuous	1 (6)
Restlessness / agitation	13 (76)
Autonomic symptoms, n (%)	
Tearing	13 (76)
Conjunctival injection	11 (65)
Feeling of sand / itching in eye	8 ( 47)
Nasal stuffiness	7 (41)
Rhinorrhea	7 (41)
Ptosis	5 (29)
Lid edema	3 (18)
Miosis	1 (06)
Migrainous symptoms, n (%)	
At least one migrainous symptom	9 (53)
Nausea	5 (29)
Vomiting	1 (6)
Photophobia	4 (24)
Phonophobia	4 (24)
Co morbid conditions	
Hypertension	4(24)
Diabetes Mellitus	3(18)
Restless Leg Syndrome	1(6)
Follow up duration (months)	
(mean,SD, range)	12.5 ± 6.7, 3–24

**Table 2:** Atypical Cases of parosysmal hemicrania

	**Case 1**	**Case 2**	**Case 3**	**Case 4**	**Case 5**
Age	34	36	24	40	42
Sex	M	F	F	M	F
Duration of illness (months)	9	14	9	6	24
Course	Chronic	Chronic	Chronic	Chronic	Chronic
Laterality	Right	Left	Left	Right	Right
Site of pain	Orb, tem,	Orb, tem, front	Orb, tem	Orb, tem, front	Orb, tem,
Character of pain	Severe, throb, nonthrob	Severe non throb	Severe throb	Severe nonthrob	Severe throb,
Interictal pain	Yes	–	–	–	–
Autonomic features	–	–	lacri, conj	conj, rhin	Conj, lacri,
Migrainous features	nau, phot,	nau,	–	nau, phot, phon	–
Restlessness	yes	yes	yes	yes	yes
Nocturnal attacks	yes	yes	–	yes	yes
Frequency of attacks ( range /day)	4–10	4–15	0–5	1–4	5–15
Duration of attacks	5–20 min	Few sec- 20 min	5–20 min	5–30 min	5–20 min
Response to indomethacin					
	Complete	Complete	Complete	Complete	Incomplete
Doses (mg/day)	150	75	150	75	225^*^
Days taken to show complete response	4	2	4	3	–
Side effects of indomethacin	pain abd,	–	Nau, vom	–	–

* = maximum tried dose.

*abd*: abdomen; *conj*: conjunctival injection; *front*: frontal; *lacri*: lacrimation; *nau*: nausea; *nonthrob*: nonthrobbing; *orb*: orbital; *phon*: phonophobia; *phot*: photophobia; *rhin*: rhinorrhea; *tem*: temporal; *throb*: throbbing; *vom*: vomiting.

### Epidemiological and clinical features of patients fulfilling the ICHD-II criteria for PH

The mean age of participants was 38 years, 53% were female. The mean illness duration was 38.2 ± 38.1 months. All patients had strictly unilateral pain. None of the patients reported bilateral or side-shifting pain. All patients reported pain in the distribution defined in the ICHD-II diagnostic criteria for PH. Orbital/retro orbital was the most common site of pain (88%). Temporal and frontal regions were two other common sites of pain. Two patients (12%) reported pain even in the neck. All patients had at least one autonomic feature as defined in the diagnostic criteria for PH. However, autonomic features were not consistent in each attack in every patient. Only six patients (35%) reported autonomic features in each or most of the attacks. In five patients (29%), cranial autonomic features were noted in less than half of the attacks. Lacrimation (76%) and conjunctival injection (65%) were the two most common cranial autonomic features. Other common cranial autonomic features were feeling of sand/itching in the eye (47%), nasal stuffiness (41%), rhinorrhea (41%), and ptosis (29%).

Most patients (88%) reported their pain as severe or very severe. Only two patients (12%) reported their pain as moderately severe. Seventy-six percent patients used the word “excruciating” to define their pains. Fifty-three percent patients reported their pain as throbbing. Interictal pain was noted in eight (47%) patients. The ipsilateral interictal pain was mild in comparison to attack pain in all patients. Most patients (7 out of 8 patients) had intermittent interictal pain. The diagnosis of hemicrania continua (HC) was not considered in these patients because of the mild and intermittent interictal pain. The frequency and duration of the attacks were in accordance with the diagnostic criteria for PH. Moreover, these all patients denied the presence of interictal pain during early periods of illness. In the subgroup analyses, we compared patients having interictal pain to the patients not having such background headache. There were statistically significant differences in two parameters (Table 3). The patients having interictal pain have a more chronic course (69.0 vs 10.7 months, p = 0.0006). The indomethacin dose requirement was also higher in patients with interictal pain (187.5 vs 113.9, p = 0.0018). There were no other differences between the groups (data not shown).

**Table 3:** A comparison between patients with interictal pain to patients without interictal pain

**Parameters**	**Interictal pain (n-8)**	**Without interictal pain (n-9)**	**p value**
Duration of illness (months)	69.0 ± 40.0	10.7 ± 6.2	0.0006^*^
Indomethacin dose (mg/day)	187.5 ± 40.1	113.9 ± 39.7	0.0018^*^

* p = <0.005: significant.

The usual duration of the attacks was between 2–30 minutes (in accordance to the ICHD-II criteria for PH) (Table 4). However, the duration of attacks varied between a few seconds to 3 hours. The longest attack duration was more than 30 min in four patients (24%). Two patients reported longest headache duration of 3 hours. The possibility of cluster headache (CH) was less likely in these patients as the overall duration of attacks (and frequency of attacks) were more compatible with the diagnosis of PH. None of the patients showed any circadian periodicity. Absence of circadian rhythmicity and periodicity (clustering) also favors the diagnosis of PH.

**Table 4:** Durations and frequency of attacks in PH

**Parameters**	**PH (n-17)**
Durations of attacks	
< 2 minutes	7 (41%)
2–30 minutes	17 (100%)
> 30 minutes	4 (24%)
Attacks frequency	
>5/day for more than half of the time	17 (100%)
maximum attack frequency	10–20/day
minimum attack frequency	0–5/day
Nocturnal attacks	6 (35%)

The ICHD-II criteria for the frequency of attacks in PH are > 5 attacks per day for more than half of the time. All included patients fulfilled this criterion. The maximum attack frequencies in these 17 patients were 10–20 per day. The minimum attack frequencies were 0–5 per day. Three patients never had frequency of more than 5 attacks per day. We described it separately as atypical PH (Table 2). Nocturnal attacks were noted in 6 patients (35%). However, none of the patients reported nocturnal preponderance or periodicity. Agitation or a sense of restlessness was present in 13 patients (76%).

There was at least one migrainous feature of nausea, or vomiting, photophobia, or phonophobia in 9 (53%) patients.

### Periodicity and chronicity of PH

PH is classified as episodic paroxysmal hemicrania (EPH) or chronic paroxysmal hemicrania (CPH) depending on the presence of a remission period. Fifteen (88%) patients had a chronic course. Fourteen of these patients had a chronic course since onset (primary chronic CPH). The headache began as EPH and transitioned to CPH in one patient. The course of headache was like primary EPH in two patients (12%).

### Response to indomethacin

A response to indometacin is considered as a *sine qua non* in PH [1]. All patients responded to indomethacin. The details of response to indomethacin are summarized in Table 5. The mean effective daily dose of indomethacin was 149 mg (range 50–225). As noted earlier, the indomethacin dose requirement was higher in patients with interictal pain (187.5 vs 113.9, p = 0.0018). Fifteen patients (88%) showed a complete response in a week (7 days). Another two patients (12%) took 1–2 weeks to show the complete response to indomethacin. None of the patient was subjected to “indo” test because of the non availability of injectable indomethacin. Eight patients (47%) were subjected to tapering off indomethacin. Only one patient was successfully weaned off the drug. The patient did not have recurrence of headaches in the next 6 months follow up. There was no prior history of episodic pattern of PH in this patient. Sudden withdrawal of indomethacin (because of the side effects of indomethacin or poor compliance) was noted in 7 patients. Withdrawal of the drugs led to a recurrence of headaches within 2–10 days.

**Table 5:** Details of indomethacin used in patients with PH

**Parameters**	**PH (n-17)**
Indomethacin dose	
Mean ± SD ( mg\daily)	149 ± 54
≤ 75 mg\daily	3 (18)
> 75–150 mg/daily	10 (59)
> 150–225 mg/daily	4 (23)
> 225 mg/daily	0 (0)
Time interval between indomethacin administration and complete response	
< 24 hrs	4 (23)
1–7 days	11 (65)
> 7 days	2 (12)
Side effects by indomethacin	
Nausea/vomiting	9 (53)
Abdominal pain	4 (23)
Diarrhea	2 (12)
Dizziness/vertigo	2 (12)
somnolence	1 (6)

Prospectively, we gave topiramate to 4 patients. Topiramate was started because of the development of side effects of indomethacin. Two patients showed a complete response to the drug. Both the patients showed complete response at the dosage of 100 mg bid.

None of the patients received a correct diagnosis before reporting to us. Migraine (65%), cluster headache (59%) and atypical facial (35%) pain were the most common diagnoses. Various drugs have been used in the past in these patients. The details were not available for each drug in each patient. However, five patients responded partly (> 50%) to either verapamil (3 patients) or lithium (2 patients). These all four patients (diagnosed previously as CH) showed a complete response to indomethacin. Frequencies, duration, pattern (without periodicity) of the attacks were also more compatible with the diagnosis of PH.

### Atypical paroxysmal hemicrania

We identified 5 patients in whom one of the features of the diagnostic criteria for PH was missing. We identified 2 patients with PH-like headaches without any cranial autonomic features. These 2 patients look like CPH, with respect to the frequency and duration of attacks and response to indomethacin. They fulfilled the ICHD-II diagnostic criteria for CPH, with the exception of autonomic symptoms. There was no better alternative diagnosis for these patients. We considered these patients as atypical or probable PH. In parallel, we identified 2 additional patients with PH-like headaches except the absence of the required number of the attacks per day to fulfill the ICHD-II criteria for PH. The ICHD-II criteria for the number of attacks per day are more than five attacks for more than half of the time. The attack frequency in these two patients was ≤ 5 / day. These 2 patients resemble CPH in respect to other features of PH according to the ICHD-II criteria. There was no better alternative diagnosis for these patients. We considered even these patients as atypical or probable PH. One patient fulfilled all the features of PH except the complete response to indomethacin. This patient showed marked improvement in frequency (5–15 /day to 0–5/day). The intensity and duration of attacks also reduced markedly. The patient had never felt such type of improvement with any drug in the past.

## Discussion

### Prevalence and other epidemiological feature

PH is considered as a rare primary headache disorder. It is suggested that cases of PH are probably overlooked or underreported [4]. PH was first described in 1974 [5]. In the first 15 yrs (up to 1989), a total 84 cases of PH were reported in the literature [6]. However, in the next 10 years (up to 1998), only 27 cases were reported [7]. In 2002, Boes and Dodick reported 74 patients with CPH (Goadsby and Lipton’s criteria) [2]. Isolated PH cases are no longer reported now days [4]. However, the literature is scarce even for larger case series. During the last 12 years, only one large case series (31 patients) has been reported [8]. These suggest that cases of PH are underreported or overlooked.

In the early years, the prevalence of PH was estimated in relation to the prevalence of CH, and it was 0.9 to 3% [6]. This estimation was done by Antonaci and Sjasstad in 1989 (within the first 15 yrs of the discovery of the first case of PH) [6]. The authors predicted “this ratio may change considerably in the foreseeable future”. After 1989, we noted at least two studies where PH was compared to CH. In 2002, Faud and Jones [9] reported 11 patients with PH. In parallel, they reported 30 patients with CH over the same period. The ratio of PH to CH was 30%. In the same way, this ratio was 15% in Zidverc-Trajkovic et al. study [10]. According to these two studies, the prevalence of PH (with respect to CH) may be more than 15% of CH. This indicates that PH is probably more common than it was anticipated earlier. Seidel et al. [11] reported 63 consecutive patients with unilateral headache not resembling migraine or CH. Only twenty-four patients received a diagnosis of primary headache disorders. Six of them had PH. Blankenburg [12] reported 8 children with PH among 628 children (1.3%) with chronic daily headache and suggested that PH is under diagnosed even in children. A possibility of misdiagnosis also exists. Initially, PH was considered as a disease of the female (2.3 to 7: 1) [4]. However, in Cittadini case series (31 patients), female preponderance was not obvious. The authors suggested that it may be because of mis-diagnosis of males with PH as cluster headache, as it is the more common with a distinct male preponderance. Our all patients were never diagnosed previously before reporting to us. This suggests that a possibility of mis-diagnosis or unawareness to PH may be very high.

Taken together, PH is probably an under diagnosed and underreported primary headache disorder. We observed 17 patients with PH (and 5 probable PH) over 6 years duration. Our case series is probably the third largest case series in the literature.

The mean age of onset (38 yrs) was comparable to other studies. In Cittadini et al. series [8], it was 37 yrs and in a review of 84 patients, mean age was 34 yrs [6]. PH is classically considered as a disease with female preponderance. However, in Cittadini et al. series [8], male outnumbered female (17 vs 14). In our series male: female was 1:1.1 (8 male vs 9 female). These two observations suggest that both male and female may be equally affected.

### Duration and frequency of attacks

Seventeen patients fulfilled the criteria for PH. The closet differential diagnosis of PH is CH [4]. Clinical characteristics for both PH and CH is similar. PH is usually distinguished from other TACs by the duration and frequency of attacks and by a response to indomethacin in patients with PH [1,4]. However, as there is overlap in the diagnostic criteria for PH and CH, a possibility of misdiagnosis always exist in patients with PH and vice-versa [8,13]. Presence of indomethacin responsive CH may further complicate the issue [13].

In our all 17 cases of (typical) PH, duration and frequency of attacks were more compatible with the diagnosis of PH. All patients had headache duration between 5–30 minutes (as defined in the ICHD-II criteria for PH). Only four patients (24%) had a few headache attacks of more than 30 minutes. In Cittadini et al. series [8], 55% patients had the longest attack duration of more than 30 min. In Boes and Dodick case series [2], the maximum attack duration was more than 60 minutes in 41% patients. A patient may have a few attacks of many hours. This indicates that a few attacks in a patient may be more than of 30 minutes (an upper limit defined in ICHD-II criteria for PH). This may create diagnostic confusion, and a misdiagnosis as CH or migraine exists if patient or physician focuses mainly on maximum attack duration. Therefore, mean or average duration of the attacks should be considered in making the diagnosis of PH and other TACs.

In the same way, the frequency of attacks is also an important point in making the diagnosis of PH. More than 5 attacks per day for more than half of the time is essential to make the diagnosis of PH. All 17 patients (typical PH) fulfilled this criterion. The maximum attack frequency was ≥ 10 / day in all 17 patients. However, we observed 2 more patients who never had 5 or more attacks in a day. Other clinical features, including response to indomethacin were according to the ICHD-II criteria for PH. We considered these two patients as atypical PH. Although ICHD-II acknowledges the presence of lower frequency in patients with PH, ≥ 5 / day for more than half of the time is must to fulfill the criteria. In review of the literature, we noted a number of patients with the maximum attack frequency of < 5 / day. The maximum attack frequency was between 2 and 5 in 37% patients in Boes and Dodick case series [2]. In Cittadini case series [8], at least three patients had a maximum attack frequency of less than five in a day. In Zidverc-Trajkovic et al. observations [10], two patients (out of eight patients) had attack duration shorter than proposed by ICHD-II criteria. These observations suggest that a few patients may never have attack frequency of more than 5 in a day.

### Cranial autonomic features

Patients with PH are required to have at least one of the cranial autonomic features according to ICHD-II criteria. Our three patients (16%) never had autonomic features. Two (4%) patients in Boes and Dodick case series [2] had no autonomic features. One patient in Cittadini case series had not any classical autonomic features and reported only a sense of ear fullness during the attacks. Maggioni [3] reported a case of episodic PH with no cranial autonomic feature and suggested a possibility of a subgroup of PH. Similar observations have also been noted in patients with CH and HC. Three to seven percent patients with CH may never have cranial autonomic features during CH attacks [14]. Various case series suggest that a large number of patients with HC may never have autonomic feature during the exacerbation of attacks [15]. Feeling of sand or itching in eye is classically described as an autonomic feature in patients with HC [15]. Our 8 patients (47%) mentioned the feeling of sand or itching in eye during the headache episodes. These observations need to be confirmed prospectively in other case series.

It is suggested that the presence of autonomic features is related to the intensity of the headaches [14]. Autonomic features are less likely to occur in patients with moderate to severe rather than excruciating attacks of pain. As the intensity of pain attacks in PH is much less than attacks of CH, absence of autonomic feature is possible even in patients with PH. ICHD-II acknowledges the diagnosis of CH in the absence of cranial autonomic features ( if patient has restlessness) [1]. The similar suggestion has been given even for making the diagnosis of HC (i.e. HC should be diagnosed even in the absence of cranial autonomic feature) [15]. Therefore, we suggest that ICHD-II should acknowledge the diagnosis of PH even in the absence of autonomic symptoms.

### Restlessness/agitation

Restlessness or agitation is classically described in patients with CH and it is one of the components of the ICHD-II diagnostic criteria for CH. It provides an alternative to make the diagnosis of CH even in the absence of autonomic feature. A few authors suggest that restlessness or agitation should also be included in the diagnostic criteria for HC [15,16]. Review of the literature suggests that restlessness or agitation may be noted in 50-90% of patients with PH. Our 76% reported restlessness or agitation. We suggest that like CH, restlessness / agitation should be included in the diagnostic criteria for PH, as it would provide alternative to make the diagnosis of PH in the absence of autonomic feature.

### Lack of complete indomethacin response

A response to indometacin is considered as a *sine qua non* in PH. Our one patient had an incomplete response to indomethacin. In Boes and Dodick case series [2], 25% (10/40) patients did not exhibit responses to indomethacin. A few other patients (6 patients) had only a partial response to indomethacin. In the other two patients, a complete response to indomethacin was not sustained.

HC is another indomethacin responsive primary headache disorder. However, recent observations indicate that the indomethacin resistant HC is a distinct possibility [15,17,18]. In an earlier review, we suggested a possibility of under reporting of HC unresponsive to indomethacin, as it is difficult to classify such patients according to the present ICHD-II classification [18]. The same possibility may exist for PH unresponsive to indomethacin and it may be the reason for underreporting of PH in the literature. In a review, we noted that CH may be wrongly diagnosed as PH if patients with CH show a response to indomethacin [13]. Therefore, a possibility exists that PH unresponsive to indomethacin (especially borderline cases) can be diagnosed as CH. As the prevalence of CH is very high than PH, a possibility of misdiagnosis of PH as CH is more likely (than CH diagnosed as PH). Patients with PH also show response to lithium, verapamil, and topiramate. Therefore, a patient will not receive the diagnosis of PH, if a patient shows a response to another drug before a trial to indomethacin. Our five patients (29%) showed a partial response to lithium and verapamil (before a trial of indomethacin), and were labeled as CH. Prospectively our 2 patients showed a response to topiramate.

### Interictal pain

Eight of 17 patients (47%) reported interictal pain. Seven of these (88%) had intermittent pain. This interparoxysmal pain was usually mild and was mainly described as discomfort in that area. The presence of interictal pain may create diagnostic confusion with HC [15]. However, “mild” and “intermittent” nature of interparoxysmal pain favors the diagnosis of PH. Moreover, frequency and duration of attacks were more compatible with the diagnosis of PH. Frequency and duration of the exacerbations in patients with HC are highly variable and duration of attacks frequently crosses the upper defined levels of PH (and CH) [15]. The exacerbations in a patient with HC vary between a few minutes to many hours (may be upto days) [15]. One patient reported continuous inteictal pain. The interictal pain was mild, and periodic exacerbations matched with the frequency and duration described for PH. Moreover, the patient did not have interictal pain during the early years of the disease.

We compared patients with interictal pain to patients with no such pain. The mean duration of illness was significantly higher in patients with interictal pain (69.0 vs 10.7 months, p = 0.0006). In the same way, patients with interictal pain required a high dose of indomethacin (188 vs 114 mg, p = 0.0018). These observations indicate that patients with longer duration of history are more likely to have interictal pain and these patients may require higher doses of indomethacin. Such type of observations was also noted in patients with CH [19]. In Marmura et al’s case series [19], patients with a long history of disease were more likely to have interictal pain. The patients with a long history of disease and interictal pain were more refractory to the therapy. Various mechanisms have been suggested for these observations. It is said that any longstanding chronic pain may lead to cortical changes in the brain and it may be responsible for the interictal pain and refractoriness to therapy [19-21]. As these patients may require higher doses of indomethacin, an early diagnosis is very important.

Taken together, our case series and review of the literature suggest that a subgroup of patients may not fulfill the all the features of ICHD-II diagnostic criteria of PH and a possibility of a subgroup of PH exist. Therefore, we suggest the inclusion of a more accommodating alternative diagnostic criteria or probable PH in the appendix section.

There are marked overlap in clinical features and therapeutic responses among TACs. Clinical features and therapeutic responses in patients with HC also overlap with TACs. Most pathophysiological studies on TACs have been done in CH. The literature is relatively sparse on PH, SUNCT, and HC. However, it is also suggested that all three TACs and HC have common pathophysiology [22]. In the absence of clear cut biological marker, it will be difficult to differentiate borderline or atypical cases of TACs and HC. In Seidel et al. [11] case series of 63 consecutive patients with unilateral headache not resembling migraine or CH, only twenty-four patients (38%) received a diagnosis of primary headache disorders. Headaches could not be classified according to present ICHD-II criteria in 49% patients. This suggests that there is need of refinement of diagnostic criteria of PH and other unilateral headache disorders.

Migraine is the best studied primary headache disorder with well defined pathophysiology. In spite of these, ICHD-II has provided an alternative diagnostic criterion in the appendix section. In the same way, there is need of alternative diagnostic criteria of various other primary headache disorders, including PH. Therefore, till we get biological marker of PH or other TACs, it will be better to have more accommodating type of criteria in the appendix section so that we can refine rare primary headache disorders in the future.

### Limitations of our observations

This is a retrospective study and a possibility of unrecognized selection bias and recall bias exists. Although we included only those patients who had normal neuroimaging, we cannot rule out secondary headaches, as full evaluation for secondary headache was not performed on each patient. In addition, we did not have the facilities to do ‘indotest’. Our observation did not have large enough numbers to reveal true differences between the typical PH (ICHD-II fulfilled) and atypical PH. Despite these limitations, our observations and review of the literature suggests that a few patients may not fulfill all the ICHD-II criteria for PH and there is need for refinement of the criteria.

## Conclusion

Present diagnostic criteria for PH are too restrictive and a subset of patients may not have all the defined features of PH. There is a need for refinement of the existing diagnostic criteria.

## Consent

Written informed consent was taken from the patients to publish the report.

## Competing interests

The authors declare that they have no competing interests.

## Authors’ contributions

Conception and design: SP, PB, AS, AT. Analysis and interpretation of the data: SP, PB, SA, AP. Literature search: SP. Drafting of the article: SP, PB, AS. Critical revision of the article for important intellectual content: AT, SA, AP. All authors read, revised and approved the final manuscript.
